# Myocardial dysfunction assessed by speckle-tracking in good-grade subarachnoid hemorrhage patients (WFNS 1–2): a prospective observational study

**DOI:** 10.1186/s13054-023-04738-6

**Published:** 2023-11-21

**Authors:** Hugues de Courson, Grégoire Chadefaux, Alexandre Loiseau, Delphine Georges, Matthieu Biais

**Affiliations:** 1 Department of Anesthesiology and Critical Care, Bordeaux University Hospital, Place Amélie Raba-Léon, Hôpital Tripode, 33000 Bordeaux, France; 2 INSERM, BPH, U1219, University of Bordeaux, 33000 Bordeaux, France; 3 INSERM, U1034, Biology of Cardiovascular Diseases, University of Bordeaux, 33600 Pessac, France

**Keywords:** Myocardial dysfunction, Subarachnoid hemorrhage, Speckle-tracking echocardiography, Neurocritical care, Tako-Tsubo, Ventricular systolic function

## Abstract

**Background:**

Cardiac complications due to non-traumatic subarachnoid hemorrhage (SAH) are usually described using classical echocardiographic evaluation. Strain imaging appears to have better sensitivity than standard echocardiographic markers for the diagnosis of left ventricular dysfunction. The aim of this study was to determine the prevalence of cardiac dysfunction defined as a Global Longitudinal Strain (GLS) ≥  − 20% in patients with good-grade SAH (WFNS 1 or 2).

**Methods:**

Seventy-six patients with good-grade SAH were prospectively enrolled and analyzed at admission for neurocritical care. Transthoracic echocardiography was performed on days 1, 3, and 7 after hemorrhage. Routine measurements, including left ventricular ejection fraction (LVEF), were performed, and off-line analysis was performed by a blinded examiner, to determine 2-, 3-, and 4-cavity longitudinal strain and left ventricular GLS. GLS was considered altered if it was ≥  − 20%, we also interested the value of ≥  − 17%. LVEF was considered altered if it was < 50%.

**Results:**

On day 1, 60.6% of patients had GLS ≥  − 20% and 21.2% of patient had GLS ≥  − 17%. In comparison, alteration of LVEF was present in only 1.7% of patients. The concordance rate between LVEF < 50% and GLS ≥  − 20% and LVEF ≥ 50% and GLS <  − 20% was 46%.

**Conclusion:**

Strain imaging showed a higher prevalence (60.6%) of left ventricular dysfunction during the acute phase of good-grade SAH (WFNS 1 or 2) than previously described.

**Supplementary Information:**

The online version contains supplementary material available at 10.1186/s13054-023-04738-6.

## Background

Subarachnoid hemorrhage (SAH) is a rare but serious condition. Cardiac complications occurring in the early phase have been well described and include cardiac biomarker release, electrocardiogram changes, or ventricular dysfunction [1]. An association between echographic myocardial damage in the early phase of SAH and the adverse outcome has been demonstrated [2, 3]. The prevalence of this damage varies from 8 to 28% depending on the study and technique used [4].

A newer ultrasound approach to measuring myocardial function, speckle tracking imaging or strain has been available for several years [5]. This technique, which is based on analysis of deformational movements of the myocardium [6] appears to be more sensitive [7, 8] and reproducible [6, 9, 10] than techniques normally used to estimate left ventricular (LV) function.

2D-strain has become indispensable in cardiology but has only poorly been studied in SAH. In 2015, Cinotti et al. found severe impairment of global longitudinal strain (GLS) in 37% of patients admitted to an intensive care unit (ICU) with a poor-grade SAH (World Federation of Neurologic Surgeons: WFNS ≥ 3), although left ventricular ejection fraction (LVEF) was impaired in only 10% of patients [11]. To date, no study has examined GLS in patients admitted with good-grade SAH (WFNS 1 or 2).

The main objective of this study was to determine the prevalence of LV myocardial dysfunction defined as GLS ≥ -20% on the first day after SAH. We hypothesized that GLS would be more sensitive than LVEF in detecting neurogenic stress cardiomyopathy.

## Materials and methods

This prospective, single-center, and observational study (ClinicalTrial.gov ID:NCT03761654) was approved by the Ethics Committee (Ile de France Research Subjects Protection Committee VI-ID-RCB:2018-A02434-51, November 21^st^,2018) and was performed in accordance with the Declaration of Helsinki. According to French law, all patients were provided with written information about the study, and their informed consent to participate was obtained.

### Patients

Inclusion criteria were all consecutive adult patients (≥ 18years) admitted to neuro-ICU because of a good-grade non-traumatic SAH, defined as WFNS grade 1–2 [12] with or without an aneurysmal cause. Non-inclusion criteria were poor echogenicity or inability to obtain useful images, cardiac history (permanent arrhythmias, malformations, surgery, ischemia, dilated cardiomyopathy, severe valvular disease), and refusal of the patient or the patient’s representative to participate in the study.

### Study design and data collection

All patients were treated according to the Neurocritical Care Society guidelines [13]. They were continuously monitored by pulse oximetry, electrocardiogram, and invasive blood pressure. The following data were recorded on days 1, 3, and 7 after cerebral bleeding:

- Blood analysis of high-sensitivity troponin-T and B-type natriuretic peptide.
- 12-lead electrocardiogram (ECG): ECG abnormalities were defined as previously described [14].
- Transthoracic echocardiography (TTE): TTEs were performed with a Vivid S6¬Æ or a Vivid S70¬Æ (GE Healthcare, Wauwatosa, WI, USA) equipped with a 2.5-MHz transducer. TTE were performed by a trained and experienced anesthesiologist who had no knowledge of the clinical data. The usual measurements (E wave, E deceleration, A wave, lateral E' waves, E/A ratio, E/E' ratio, Aortic velocity time integral, cardiac output, lateral S' wave, tricuspid regurgitation velocity, inferior vena cava diameter) and LVEF by Simpson's biplane method were collected. LVEF impairment was defined as < 50%. Echographic cine loops were also recorded in apical 2-, 3-, and 4-cavity views for offline analysis of the strain.

Offline analysis was performed using EchoPAC® software (GE Healthcare) by a single-blinded investigator. The longitudinal strain was measured in 2-, 3, and 4 cavities to determine the GLS of the LV. An altered strain was defined as ≥ -20% [15]. We were also interested in the strain threshold of ≥ -17%, which appears to be associated with increased mortality in SAH population [3].

### Study aim

The main objective was to determine the prevalence of LV dysfunction, defined as a GLS ≥  − 20% on the first day after SAH.

### Statistical methodology

Continuous data were described in terms of mean(standard deviation) or median [interquartile range] according to their distribution. Categorical data were described by their count (percentage). Correlation between echographic data was calculated considering repeated measurements [16]. The concordance rate was estimated from a four-quadrant plot showing LVEF and GLS. Continuous variables were compared using the Wilcoxon test. All analyses were performed with R version 4.0.2 (June 2020).

## Results

### Study population

During the study period, 252 patients were eligible (Additional file 1) and 74 patients (46 females) were analyzed. Patients’ characteristics are listed in Additional file 2. Thirty-three patients had a complete record of longitudinal strain in the 2, 3, and 4 cavities on day-1, which allowed measurements of GLS.

### Echographic data

**Fig. 1:**
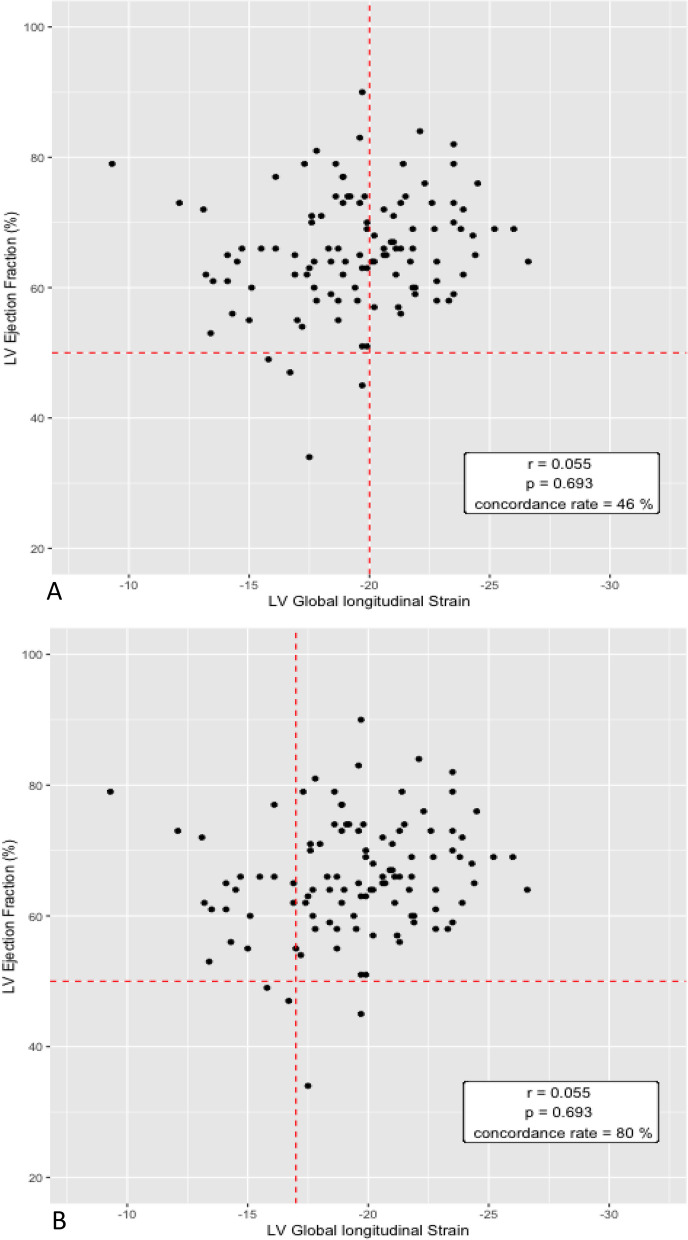
Relationship between left ventricular ejection fraction (LVEF) and global longitudinal strain (GLS). **A** Strain threshold of − 20. **B** Strain threshold of − 17

Hemodynamic and echographic data for each day are shown in Table 1. There was no difference in the mean GLS between men and women, respectively, − 19 [-21; − 18] and − 20 [-22; − 18]; *p* = 0.2. On day 1, 60.6% of patients had GLS ≥  − 20% and the proportion of patients with GLS ≥  − 17% was 21.2%. Alteration of LVEF was present in 1.7% of patients on day-1. LVEF and GLS were not correlated (*p* = 0.693). The concordance rate between LVEF < 50% and GLS ≥ -20% and LVEF ≥ 50% and GLS < -20% was 46% (51/110). When considering a strain threshold of − 17, the concordance rate rises to 80% (Fig. 1). There appears to be no variation between days for LVEF, GLS, 4C-LS (Additional file 3). The evolution of GLS during follow-up in patients with normal or altered GLS at day 1 is shown in Additional file 4.

**Table 1:** Hemodynamic and echographic data

	Day 1		Day 3		Day 7	
	n		n		n	
Heart rate (BPM)	60	70 [62–75]	70	70 [65–75]	67	73 [63–80]
Mean arterial pressure (mmHg)	61	100 [88–110]	70	100 [92–112]	68	100 [93–110]
Cardiac output (L/min)	58	5.1 [4.1–6.1]	70	4.9 [3.9–5.5]	65	4.8 [4.1–5.8]
Systemic vascular resistance (dyn·s/cm^5^)	58	1607 [1318–2042]	69	1866 [1314–2094]	65	1606 [1366–2095]
E wave (cm/s)	62	80 [68–90]	71	79 [67–94]	68	76 [62–87]
E deceleration (ms)	60	213 [166–259]	71	201 [165–223]	68	199 [176–263]
A wave (cm/s)	62	70 [23–85]	71	67 [60–83]	68	71 [58–81]
E/A ratio	60	1.1 [0.9–1.4]	71	1.1 [0.9–1.4]	68	1.1 [0.9–1.3]
E’ wave (cm/s)	62	12 [10–14]	71	11 [9–13]	67	12 [9–14]
E/E’ ratio	62	7 [6–8]	71	7 [6–9]	67	7 [5–8]
LVEF (%)	60	65 [60–71]	71	66 [62–72]	68	66 [59–74]
LVEF < 50% (n)	60	1 (1.7)	71	1 (1.4)	68	4 (5.9)
Aortic VTI (cm)	60	24 [21–27]	71	23 [21–27]	67	24 [21–26]
S’ (cm/s)	59	17 [15–19]	71	16 [14–19]	66	17 [15–20]
TRV (m/s)	59	2.3 [1.9–2.6]	69	2.2 [1.7–2.6]	65	2.2 [1.7–2.5]
Inferior vena cava diameter (cm)	58	1.4 [1.0–1.7]	65	1.3 [0.9–1.7]	61	1.2 [0.9–1.7]
2-Cavity Longitudinal Strain	45	− 20 [− 23– − 18]	46	− 19 [− 22– − 17]	48	− 19 [− 21– − 16]
3-Cavity Longitudinal Strain	49	− 19 [− 22– − 16]	53	− 21 [− 19– − 17]	50	− 20 [− 22– − 17]
4-Cavity Longitudinal Strain	55	− 19 [− 21– − 16]	69	− 19 [− 21– − 17]	62	− 19 [− 21– − 17]
Global Longitudinal Strain	33	− 20 [− 23– − 17]	38	− 20 [− 21– − 18]	39	− 20 [− 22– − 18]
Global Longitudinal Strain ≥ − 20 (n)	33	20 (60.6)	38	21 (55.3)	39	22(56.4)
4C- Global Longitudinal Strain ≥ − 20 (n)	55	34 (61.8)	69	39 (56.5)	62	40 (64.5)
Global Longitudinal Strain ≥ − 17 (n)	33	7 (21.2)	38	6 (15.8)	39	7 (17.9)
4C- Global Longitudinal Strain ≥ − 17 (n)	55	20 (36.4)	69	15 (21.7)	62	16 (25.8)

n: available data

Values are median [25th to 75th percentile] or Values are numbers (percentage)

BPM: Beats Per Minute; LVEF: Left Ventricular Ejection Fraction; VTI: Velocity Time Integral; TRV: Tricuspid Regurgitation Velocity

### Biomarkers and electrocardiographic data

Troponin was elevated in 50% of patients on day-1, in 48% on day-3, and in 37% on day-7, with median values of 19ng/L[12–58], 13ng/L[8–43], and 14ng/L[9–75], respectively. BNP elevation was present in 98% of patients on day-1, in 92% on day-3, and in 78% on day-7, with median values of 114pg/mL [45–225], 81pg/mL [30–149], and 45pg/mL [22–76], respectively. The overall relationships between troponin and GLS and between troponin and LVEF were very weak (*r* = 0.136; *p* = 0.362 and *r* = 0.06; *p* = 0.528, respectively). ECG abnormalities were present in 52% of patients at day-1.

### Intra- and interobserver variability

The reproducibility of GLS measurements was tested before the study. GLS were measured twice in six patients by the same observer(GC) and a second observer(DG). The mean difference was calculated and divided by the mean of the two values. For GLS measurements, intraobserver reproducibility was 4.0 ± 3.7% and interobserver reproducibility was 5.3 ± 5.9%.

## Discussion

This study showed that on day 1 after SAH, more than 60% of patients had GLS ≥  − 20%, and the proportion of patients with GLS ≥  − 17% was 21.2%. In comparison, alteration of LVEF was present in only 1.7% of patients on day-1.

### Markers of left ventricular injury

The prevalence of LV dysfunction during SAH varies from 8 to 28% depending on the study and technique used [4, 17]. Cinotti et al. were the first to investigate the contribution of GLS to the diagnosis of stress cardiomyopathy during severe SAH [11]. In poor-grade SAH (WFNS ≥ 3), they found GLS impairment, in 37% of patients, although LVEF impairment occurred in only 10% on day 1. These results were confirmed in 2020 by Kagiyama et al., who found a GLS >  − 17% in 24% of patients with SAH of any grade, compared with 9% of patients with LVEF < 50% [3]. In this study, GLS >  − 17% was an independent risk factor for in-hospital mortality. In our study, we focused on the good-grade SAH population. Altered GLS was defined as GLS ≥  − 20% and affected 60.6% of patients at day 1, compared with 1.7% with LVEF < 50%. In addition, we found 21.2% of patients with GLS ≥  − 17%. Our results showed a higher prevalence of LV dysfunction during SAH than in previous studies, although our population was less severe. This could be explained by a better sensitivity of the strain method compared with the usual echocardiographic markers, and by a different definition of altered GLS than in the two previous studies.

### Strain ranges

The range of normal strain values remains under discussion. In a cohort of 549 healthy volunteers, the value of normal GLS of left ventricle was − 22.5 ± 2.7% [18]. In ICU, higher values are suggested to define impaired GLS [19]. In our study, we decided to take a GLS value ≥  − 20% as the pathological limit. We also analyzed the value of − 17, which appears to be associated with increased mortality in the population SAH [3].

### Study limitations

Our study has some limitations. Strain measurement was only available in 33 patients for the GLS and in 55 patients for the 4C-LS and we did not have a reference cardiac ultrasound in our patients. Although the operator measuring GLS was blinded to LVEF measurements, the assessor was not blinded to visual LVEF since the strain is measured on ultrasound cine loops.

## Conclusion

Strain imaging reveals a higher prevalence of LV dysfunction during good-grade SAH (WFNS 1 or 2) than usual echographic markers. Early detection of patients with altered strain should allow initiation of short- and medium-term echocardiographic monitoring. Further studies are needed to determine the impact of strain alteration in this population and to propose individualized management.

### Supplementary Information

**Additional file 1**: Flow Chart of patients screened and included.

**Additional file 2**: Patient’s Characteristics.

**Additional file 3**: Evolution of the main echocardiographic markers over time. **A** Daily variations in left ventricular ejection fraction (LVEF) in the study population. **B** Daily variations in global longitudinal strain (GLS) in the study population. **C** Daily variations in global longitudinal strain (GLS) in patients with GLS ≥  − 20% at least once. **D** Daily variations in 4-cavity longitudinal strain (4C-LS) in patients with 4C-LS ≥  − 20% at least once. Gray lines represent individual trajectories of each parameter. Blue lines represent the median trajectory of each parameter. Interquartile ranges are symbolized by the red areas.

**Additional file 4**: Evolution of GLS (Global Longitudinal Strain) during follow-up among patients with at least two Global Longitudinal Strain measurements.

## Data Availability

All data generated or analyzed during this study are included in this published article.

## Abbreviations

ECG: Electrocardiogram
GLS: Global longitudinal strain
LV: Left ventricular
LVEF: Left ventricular ejection fraction
SAH: Subarachnoid hemorrhage
TTE: Transthoracic echocardiography
WFNS: World Federation of Neurologic Surgeons
