# Supplementary figures and images for: Myocardial dysfunction assessed by speckle-tracking in good-grade subarachnoid hemorrhage patients (WFNS 1–2): a prospective observational study
Source: Crit Care. 2023 Nov 21;27:455. doi: 10.1186/s13054-023-04738-6 (PMC10664298; doi:10.1186/s13054-023-04738-6)

**Additional File 1 :** Flow Chart


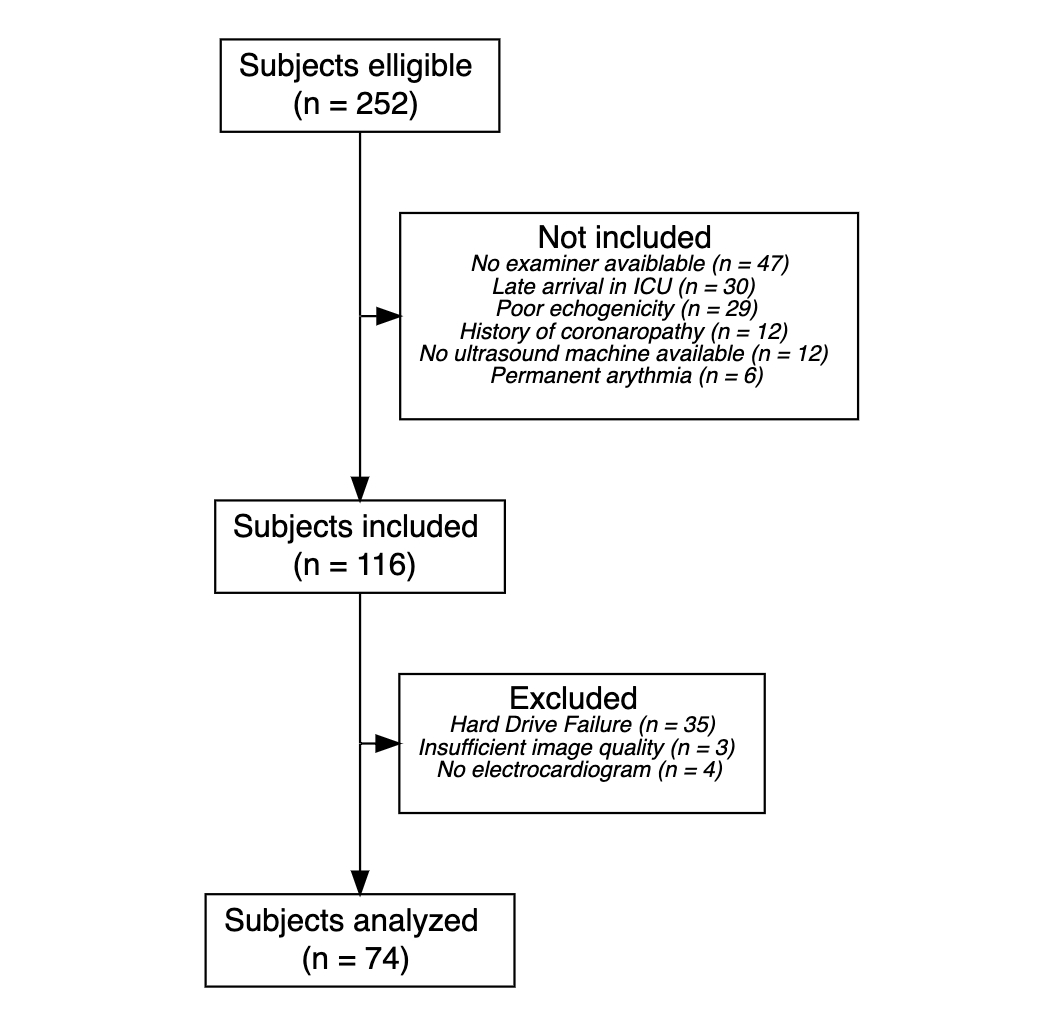

Supplement: Supplementary file 1 — Additional file 1: Flow Chart of patients screened and included. [file 13054_2023_4738_MOESM1_ESM.docx]
