# Supplementary material for: Myocardial dysfunction assessed by speckle-tracking in good-grade subarachnoid hemorrhage patients (WFNS 1–2): a prospective observational study
Source: Crit Care. 2023 Nov 21;27:455. doi: 10.1186/s13054-023-04738-6 (PMC10664298; doi:10.1186/s13054-023-04738-6)
Supplement: Supplementary file 2 — Additional file 2: Patient’s Characteristics. [file 13054_2023_4738_MOESM2_ESM.docx]

| **Additional File 2.** Patient’s Characteristics (n = 74) | |
| --- | --- |
|  |  |
| Age (years) | 53 [45– 63] |
|  |  |
| Sex |  |
| - Male | 28 (38) |
| - Female | 46 (62) |
|  |  |
| Body mass index (kg/m^2^) | 24.1 [21.6 – 28.1] |
|  |  |
| SAPS 2 | 17 [15 – 21] |
| Day of admission | 2 [1 – 2] |
| WFNS score |  |
| - 1 | 59 (80) |
| - 2 | 15 (20) |
|  |  |
| Fisher score |  |
| - 1 | 5 (7) |
| - 2 | 13 (18) |
| - 3 | 26 (35) |
| - 4 | 30 (40) |
|  |  |
| History of high blood pressure | 20 (27) |
|  |  |
| Tobacco use | 26 (35) |
|  |  |
| Cerebral vasospasm | 11 (15) |
|  |  |
| Re-bleeding | 2 (3) |
|  |  |
| Cerebral salt wasting syndrome | 37 (50) |
|  |  |
| Death | 2 (3) |
|  |  |

*Values are median [25^th^ to 75^th^ percentile], or numbers (percentage).*

*SAPS 2: Simplified Acute Physiology Score 2; WFNS score: World Federation of Neurosurgical Societies score.*
