# Supplementary material for: Myocardial dysfunction assessed by speckle-tracking in good-grade subarachnoid hemorrhage patients (WFNS 1–2): a prospective observational study
Source: Crit Care. 2023 Nov 21;27:455. doi: 10.1186/s13054-023-04738-6 (PMC10664298; doi:10.1186/s13054-023-04738-6)
Supplement: Supplementary file 3 — Additional file 3: Evolution of the main echocardiographic markers over time. A Daily variations in left ventricular ejection fraction (LVEF) in the study population. B Daily variations in global longitudinal strain (GLS) in the study population. C Daily variations in global longitudinal strain (GLS) in patients with GLS ≥ − 20% at least once. D Daily variations in 4-cavity longitudinal strain (4C-LS) in patients with 4C-LS ≥ − 20% at least once. Gray lines represent individual trajectories of each parameter. Blue lines represent the median trajectory of each parameter. Interquartile ranges are symbolized by the red areas. [file 13054_2023_4738_MOESM3_ESM.docx]

**Additional File 3. Evolution of the main echocardiographic markers over time.**

- **A:** Daily variations in left ventricular ejection fraction (LVEF) in the study population
- **B**: Daily variations in global longitudinal strain (GLS) in the study population
- **C**: Daily variations in global longitudinal strain (GLS) in patients with GLS ≥ -20% at least once
- **D**: Daily variations in 4-cavity longitudinal strain (4C-LS) in patients with 4C-LS ≥ -20% at least once

Gray lines represent individual trajectories of each parameter. Blue lines represent the median trajectory of each parameter. Interquartile ranges are symbolized by the red areas

**
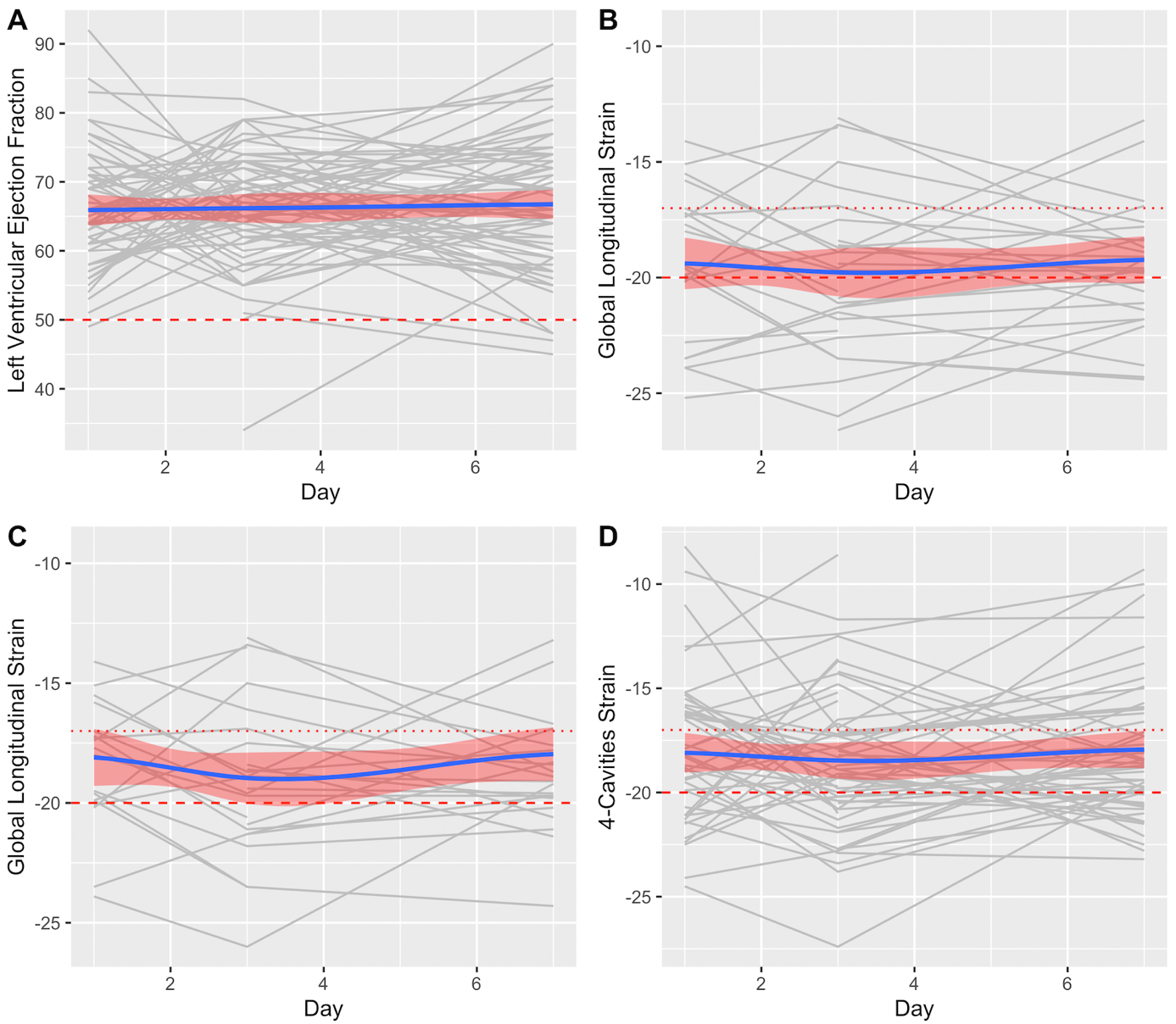
**
