# Supplementary material for: Myocardial dysfunction assessed by speckle-tracking in good-grade subarachnoid hemorrhage patients (WFNS 1–2): a prospective observational study
Source: Crit Care. 2023 Nov 21;27:455. doi: 10.1186/s13054-023-04738-6 (PMC10664298; doi:10.1186/s13054-023-04738-6)
Supplement: Supplementary file 4 — Additional file 4: Evolution of GLS (Global Longitudinal Strain) during follow-up among patients with at least two Global Longitudinal Strain measurements. [file 13054_2023_4738_MOESM4_ESM.docx]

**Additional File 4 : Evolution of GLS (Global Longitudinal Strain) during follow-up among patients with at least two Global Longitudinal Strain measurements**

- **A : Patients with normal GLS at day 1**

|  | | **No GLS deterioration during follow-up** | **GLS deterioration during follow-up** |
| --- | --- | --- | --- |
| **Normal GLS**  **at day 1** | **GLS day 1 < -20**  **n=10** | 60% | 40% |
|  | **GLS day 1 < -17**  **n=20** | 80% | 20% |

- **B : Patients with altered GLS at day 1**

|  | | **No GLS improvement during follow-up** | **GLS improvement during follow-up** |
| --- | --- | --- | --- |
| **Altered GLS**  **at day 1** | **GLS day 1 ≥ -20**  **n=15** | 60% | 40% |
|  | **GLS day 1 ≥ -17**  **N5** | 40% | 60% |
